# Bacterial Inclusion Bodies Contain Amyloid-Like Structure 

**DOI:** 10.1371/journal.pbio.0060195

**Published:** 2008-08-05

**Authors:** Lei Wang, Samir K Maji, Michael R Sawaya, David Eisenberg, Roland Riek

**Affiliations:** 1 Structural Biology Laboratory, The Salk Institute, La Jolla, California, United States of America; 2 Howard Hughes Medical Institute, University of California, Los Angeles, Los Angeles, California, United States of America; 3 University of California, Los Angeles–Department of Energy (UCLA-DOE) Institute for Genomics and Proteomics, University of California, Los Angeles, Los Angeles, California, United States of America; 4 Laboratory of Physical Chemistry, Swiss Federal Institute of Technology (ETH), Zurich, Switzerland; University of California, San Francisco, United States of America

## Abstract

Protein aggregation is a process in which identical proteins self-associate into imperfectly ordered macroscopic entities. Such aggregates are generally classified as amorphous, lacking any long-range order, or highly ordered fibrils. Protein fibrils can be composed of native globular molecules, such as the hemoglobin molecules in sickle-cell fibrils, or can be reorganized β-sheet–rich aggregates, termed amyloid-like fibrils. Amyloid fibrils are associated with several pathological conditions in humans, including Alzheimer disease and diabetes type II. We studied the structure of bacterial inclusion bodies, which have been believed to belong to the amorphous class of aggregates. We demonstrate that all three in vivo-derived inclusion bodies studied are amyloid-like and comprised of amino-acid sequence-specific cross-β structure. These findings suggest that inclusion bodies are structured, that amyloid formation is an omnipresent process both in eukaryotes and prokaryotes, and that amino acid sequences evolve to avoid the amyloid conformation.

## Author Summary

Protein aggregation is a process by which identical proteins self-associate into imperfectly ordered macroscopic entities. Such aggregates are associated with several pathological conditions in humans, including Alzheimer disease, Parkinson disease, and diabetes type II. Furthermore, protein aggregation is a major concern in the biotechnological production of recombinant proteins and the storage of proteins, and is a central mechanism of protein folding. In general, two classes of protein aggregates are classified: first, highly ordered aggregates can be composed of native globular molecules, such as the hemoglobin molecules in sickle-cell fibrils, or reorganized into β-sheet–rich aggregates, termed amyloid-like fibrils; and second, amorphous aggregates that lack any long-range order. Here, we demonstrate that bacterial inclusion bodies, which have been believed to be made up of amorphous aggregates, are in fact amyloid-like, comprising cross-β structure that is dependent on amino-acid sequence. These findings suggest that inclusion bodies are structured, that amyloid formation is a process present in both eukaryotes and prokaryotes, that amino acid sequences can evolve to avoid the amyloid conformation, and that there might be no amorphous state of a protein aggregate.

## Abstract 3

Bacterial inclusion bodies that have been classified as amorphous protein aggregates are in fact structured, comprising a cross-β-sheet structure reminiscent of amyloid fibrils.

## Introduction

The conversion of peptides and proteins into aggregates is associated with several dozen pathological conditions in humans, including Alzheimer disease, Parkinson disease, and diabetes type II [1–3], and it is also of major concern in biotechnology [4]. Numerous disease-associated aggregates are composed of elongated filaments, termed amyloid-like fibrils, often comprising intermolecular and in-register β-sheets parallel to the fibril axis [5–11]. Amyloid fibrils bind thioflavin T (Thio T) [12] and show birefringence upon Congo red (CR) staining, which is indicative of repetitive structure [13]. Amyloid fibrils, as highly ordered aggregates, have traditionally been distinguished from amorphous aggregates, such as protein precipitates and bacterial inclusion bodies [14,15]. The latter present widespread problems in biotechnology [16].

The deposition of protein products into electron-dense, apparently amorphous inclusion bodies occurs often during high-level expression of heterologous and some endogenous genes in Escherichia coli [17,18]. The aggregation is probably caused by the high local concentration of nascent polypeptides emerging from ribosomes [4], which are ineffectively protected from aggregation by the insufficient number of chaperones present during recombinant overexpression, or by the lack of eukaryotic chaperones [19]. Although inclusion bodies have been classified as amorphous aggregates, they are not just clusters of misfolded proteins that stick to each other through nonspecific hydrophobic contacts [14–16]. Rather, they are often enriched in β-sheet structure [20–22], bind amyloid-tropic dyes [21], have a seeding capacity reminiscent of amyloids [21], form homogeneous aggregates without cross-aggregation [23], and display aggregation propensities strongly affected by mutations [24]. Here, we studied in detail the structure of in vivo-derived bacterial inclusion bodies of three proteins and show that all three inclusion bodies studied are amyloid-like, comprising amino acid sequence-specific cross-β structure.

## Results

**Figure 1:**
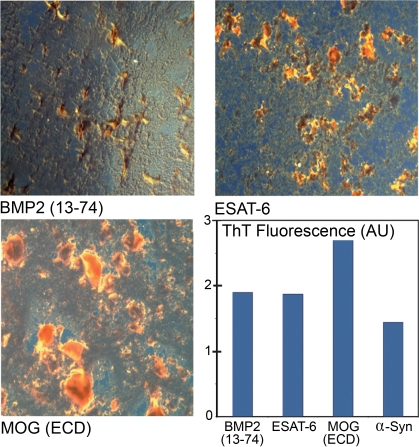
Amyloid-Like Character of Three Bacterial Inclusion Bodies CR birefringence and Thio T binding of inclusion bodies are indicative of amyloid formation. Under cross-polarized light with 10× magnification, CR birefringence is observed for BMP2(13–74), ESAT-6, and MOG(ECD). Larger images together with the corresponding pictures from the bright-field microscope are shown in Figure S1. The histogram shows the relative Thio T binding of BMP2(13–74), ESAT-6, and MOG(ECD) in comparison to aged α-synuclein (α-Syn) fibrils. The intensity of the Thio T fluorescence is shown in arbitrary units (AU).

**Figure 2:**
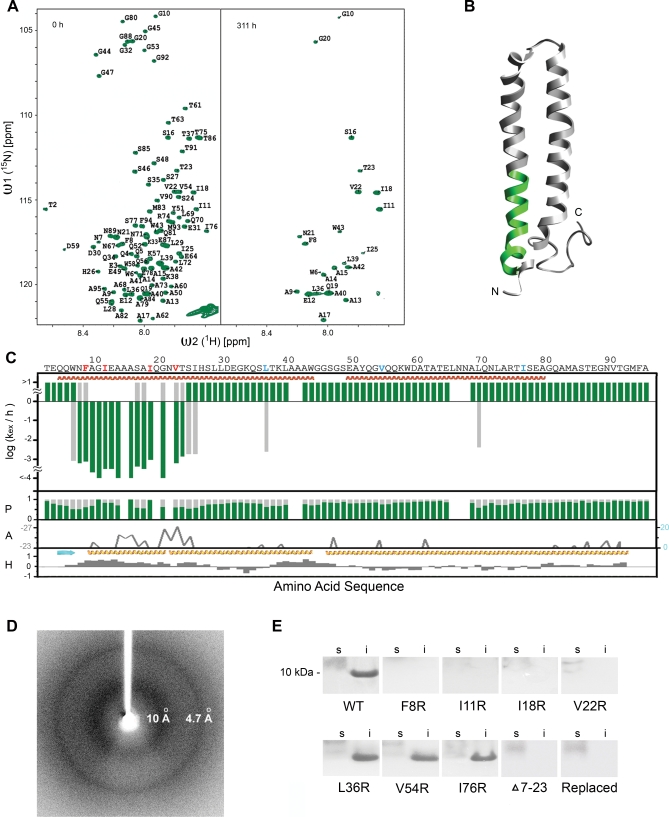
The Short Segment of Residues 7–23 of ESAT-6 Forms Cross-β-Sheet Structure in Inclusion Bodies of E. coli (A) Fast [^15^N,^1^H]-HMQC spectra of dissolved, monomeric ^15^N-labeled ESAT-6 in d_6_-DMSO containing 0.05 % TFA and 25 mM DTT, corresponding to fully protonated inclusion bodies (left), and to inclusion bodies exchanged for 311 h in D_2_O (right). After 311 h, many cross peaks show a complete loss of intensity indicative of fast exchange. In contrast, cross peaks of residues 8–25 and 36–43 are still present, indicative of slow exchange. Sequence-specific chemical-shift assignment of protein backbone amide cross peaks are indicated by a single-letter amino acid code and the corresponding residue number. (B) Ribbon representation of the 3-D structure of soluble ESAT-6 in complex with CFP-10 [25]. The green-colored segment corresponds to residues 7–23, which experiences slow exchange in inclusion bodies as shown in (C). (C) Plots of the observed exchange rates *k*_ex_/h, the relative population *P*(F) of the two exchange regimes observed with *P* = 1 for 100% occupancy and *P* = 0 for 0% occupancy, and the predictions of aggregation-prone segments against the amino acid sequence of ESAT-6. The exchange rates of the major population are colored green. If the minor population is present more than 1/3, the corresponding exchange rates are shown in grey. Although some of the residues 36–43 show slow exchange in the HMQC spectrum in (A), their slow-exchanging population is present less than 1/3 and hence not shown in (C). In the third plot of (C) labeled with an “A,” predicted aggregation-prone segments of ESAT-6 are shown using two distinct algorithms: 3DPROFILE [33] in gray, and TANGO [32] (the latter is not shown, since no aggregation-prone segment was predicted). For 3DPROFILE, predictions are shown for segments having energies ≤ −23 kcal/mol. For both algorithms, outstanding relative values (≤ −23 in 3DPROFILE, and >0 in TANGO) within a segment of several amino acid residues are indicative of an aggregation-prone segment. The secondary structures of the soluble conformation shown in (B) are highlighted in red for helix and blue for β-sheet, respectively. The secondary structural elements predicted by the software Jpred [53] are highlighted by cyan arrows for β-sheet conformation and a yellow helix for helical structure, respectively. An amino-acid sequence-resolved hydrophobicity score plot calculated by the software ProtScale [54,55] is shown at the lowest panel labeled with “H,” with positive values indicative of hydrophobicity. (D) X-ray diffraction of inclusion bodies of ESAT-6. The two reflections at 4.7 Å and approximately 10 Å consistent with cross-β-sheet structure are labeled. (E) Mutagenesis of ESAT-6 and the influence of amino acid substitutions in the formation of inclusion bodies. Coomassie-stained SDS-polyacrylamide gels were obtained from soluble (s) and insoluble (i) fractions of lysates of E. coli cells expressing wild-type ESAT-6 (WT) or ESAT-6 variants as indicated (i.e., point mutations are indicated by a one-letter code, Δ7–23 stands for the deletion variant, which lacks the slow-exchanging residues 7–23, and “replaced” stands for the mutant for which residues 7–23 were replaced with the helical segment of residues 25–41 of ESAT-6. The 10-kDa molecular weight standard is labeled. The variants that are present in the insoluble fraction are colored blue in the amino acid sequence of (C), and the single amino acid residue variants that are absent in the insoluble fractions are colored red in the amino acid sequence of (C), respectively.

**Figure 3:**
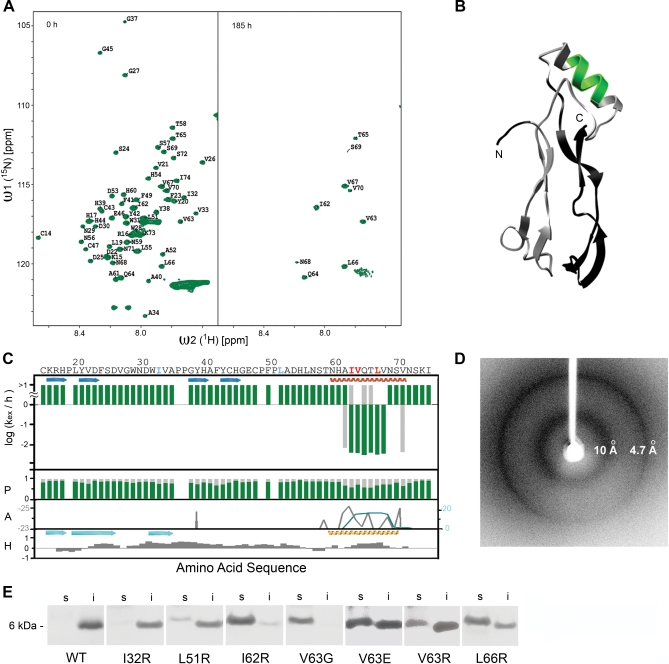
The Short Segment of Residues 62–67 of BMP2(13–74) Forms Cross-β Structure in Inclusion Bodies of E. coli (A) Fast [^15^N,^1^H]-HMQC spectra of homogenously ^15^N-labeled monomeric BMP2(13–74) in d_6_-DMSO containing 0.05 % TFA and 25 mM DTT, corresponding to fully protonated inclusion bodies (left), and to inclusion bodies that exchanged for 185 h in D_2_O (right). After 185 h, many cross peaks show a complete loss of intensity, indicative of fast exchange. In contrast, cross peaks of residues 62–70 are still present, indicative of slow exchange. (B) Ribbon representation of the 3-D structure of soluble BMP2. The green-colored segment corresponds to residues 62–67 that comprise slow exchange in inclusion bodies as shown in (A) and (C). The protein fragment BMP2(13–74) comprising residues 13–74 is highlighted in grey. The N-terminal 12 residues and the C-terminal 40 residues of BMP2 are colored black. (C) Plots of the observed exchange rates *k*_ex_/h, the relative population *P*(F) of the two exchange regimes observed (Figure S2), and the predictions of aggregation-prone segments against the amino acid sequence of BMP2(13–74). The exchange rates of the major population are colored green. If the minor population is present more than 1/3, the corresponding exchange rates are shown in grey. In the third plot of (C) labeled with “A,” predicted aggregation-prone segments of BMP2(13–74) are shown using two algorithms: 3DPROFILE [33] in gray and TANGO [32] in blue. Predictions of aggregation are shown for segments having energies ≤ −23 kcal/mol from 3DPROFILE, and values >0 from TANGO. The secondary structures of the soluble conformation shown in (B) are highlighted in red for helix and blue for β-sheet, respectively. The secondary structural elements predicted by the software Jpred [53] are highlighted by cyan arrows for β-sheet conformation and a yellow helix for helical structure, respectively. An amino acid sequence-resolved hydrophobicity score plot calculated by the software ProtScale [54,55] is shown at the bottom, labeled with “H,” with positive values indicative of hydrophobicity. (D) X-ray diffraction of inclusion bodies of BMP2(13–74), indicative of cross-β-sheet structure. (E) Mutagenesis of BMP2(13–74). Coomassie-stained SDS-polyacrylamide gels were obtained from soluble (s) and insoluble (i) fractions of lysates of E. coli cells expressing wild-type BMP2(13–74) (WT) or variants as indicated. The identities of the bands were verified by mass spectrometry analysis (see Material and Methods). The 6-kDa molecular weight standard is labeled. The same nomenclature, labeling, and layout are used as in Figure 2.

**Figure 4:**
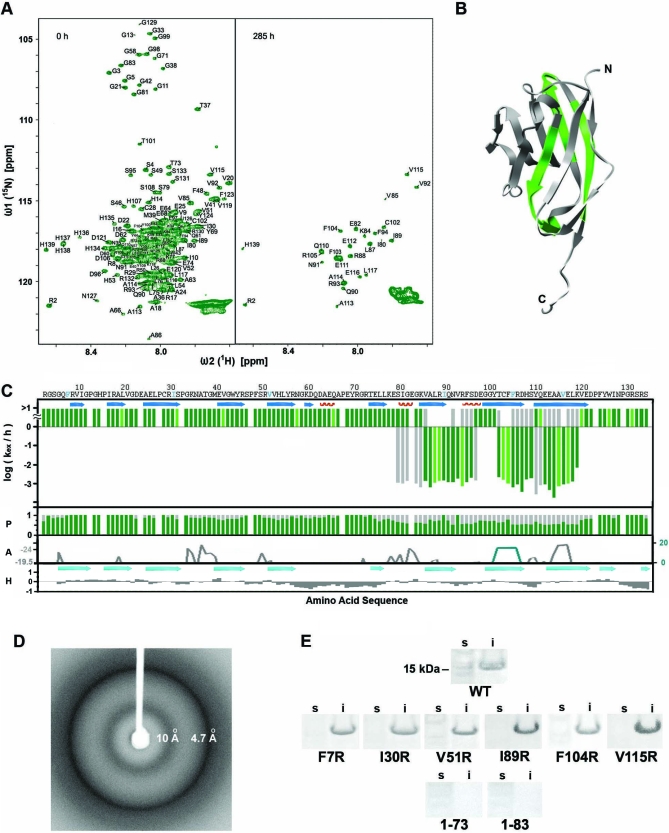
The Segments of Residues 85–95, 101–108, and 111–118 of MOG(ECD) Form a Cross-β-Sheet Structure in Inclusion Bodies of E. coli. (A) Fast [^15^N,^1^H]-HMQC spectra of homogenously ^15^N-labeled monomeric MOG(ECD) in d_6_-DMSO containing 0.05 % TFA and 25 mM DTT, corresponding to fully protonated inclusion bodies (left), and to inclusion bodies that exchanged for 285 h in D_2_O (right). After 285 h, many cross peaks show a virtually complete loss of intensity, indicative of fast exchange. In contrast, a set of cross peaks labeled by their corresponding amino acid residue number are still present, indicative of slow exchange. (B) Ribbon representation of the 3-D structure of soluble MOG(ECD) [28]. The green-colored segments correspond to residues 85–95, 101–108, and 111–118, which comprise slow exchange in inclusion bodies as shown in (C). (C) Plots of the observed exchange rates *k*_ex_/h, the relative population *P*(F) of the two exchange regimes observed, and the predictions of aggregation-prone segments against the amino acid sequence of MOG(ECD). The exchange rates of the major population are colored green. If the minor population is present more than 1/3, the corresponding exchange rates are shown in grey. Because of the size of the protein, considerable overlap is observed in the DMSO spectrum (see [A]), making the analysis of the exchange rates of some residues difficult. However, most of these overlap problems could be resolved by the assumption that sequential neighboring residues show a similar extent of exchange. The exchange rates that have been extracted following this procedure are colored in light green. In the third plot of (C), predicted aggregation-prone segments of MOG(ECD) are shown using two algorithms: 3DPROFILE [33] in gray and TANGO [32] in blue. Predictions of aggregation are shown for segments having energies ≤ −19.5 kcal/mol from 3DPROFILE, and values >0 from TANGO. The secondary structures of the soluble conformation shown in (B) are highlighted in red for helix and blue for β-sheet, respectively. The secondary structural elements predicted by the software Jpred [53] are highlighted by cyan arrows for β-sheet conformation and a yellow helix for helical structure, respectively. An amino acid sequence-resolved hydrophobicity score plot calculated by the software ProtScale [54,55] is shown at the bottom labeled with “‘H,” with positive values indicative of hydrophobicity. (D) X-ray diffraction of inclusion bodies of MOG(ECD). The two observed bands at 4.7 Å and approximately 10 Å indicative of cross-β-sheet structure are labeled. (E) Mutagenesis of MOG(ECD) and the influence of amino acid substitutions in the formation of inclusion bodies. Coomassie-stained SDS-polyacrylamide gels were obtained from soluble (s) and insoluble (i) fractions of lysates of E. coli cells expressing wild-type MOG(ECD) (WT) or variants as indicated. The 15-kDa molecular weight standard is labeled. The variants that are present in the insoluble fraction are colored blue in the amino acid sequence of (C). The same nomenclature, labeling, and layout are used as in Figure 2.

To elucidate structural details of inclusion bodies of E. coli (Figures 1–4) and compare them with the structure of amyloid fibrils, three inclusion body-forming proteins with distinctive native folds (i.e., β-sheet, α-helix, and mixed α-helix/β-sheet, with and without disulfide bridges) were chosen to cover the fold universe to a certain degree: (1) The α-helical, early secreted antigen 6-kDa protein (ESAT-6, with Protein Data Bank [pdb] accession code 1wa8; Figure 2B) from Mycobacterium tuberculosis folds only in complex with its protein partner CFP-10 (culture filtrate protein, 10 kDa) both in vitro and when overexpressed in E. coli [25]. (2) The mixed α-helical and β-sheet fragment residues 13–74 of the secretory human bone morphogenetic protein-2 (BMP2, with pdb accession code 3bmp) (Figure 3B) [26,27]. BMP2(13–74) is unable to fold in E. coli because it lacks the essential structural segment of residues 75–114, as well as the ability to form disulfide links in the cytoplasm of E. coli. (3) The β-sheet comprising the extracellular domain (ECD) of the human membrane protein myelin oligodendrocyte glycoprotein (MOG(ECD), with pdb accession code 1pko), having one disulfide bridge (Figure 4B) [28,29]. The inclusion body formation of MOG is attributed to the reducing environment of the E. coli cytoplasm.

### Inclusion Bodies of ESAT-6 Are Composed of Ordered β-Sheet Structure

All three proteins studied are present in the insoluble fraction of E. coli upon overexpression (Figures 2E, 3E, and 4E) and form homogeneous inclusion bodies as expected (unpublished data). The purified inclusion bodies of all three proteins bind Thio T to a similar or greater extent than aged amyloid fibrils of α-synuclein (Figure 1) and produce strong birefringence upon CR staining (Figures 1 and S1). These findings indicate that inclusion bodies of all three proteins studied comprise highly ordered structures reminiscent of amyloid-like fibrils.

We found, furthermore, that inclusion bodies of all three proteins contain sequence-specific positions of regular secondary structure, by measuring quenched hydrogen/deuterium (H/D) exchange with solution nuclear magnetic resonance (NMR) [9,30] (see also Materials and Methods). This technique allows the identification of solvent-protected backbone amide protons involved in hydrogen bonds. In the case of ESAT-6, the [^15^N,^1^H]-correlation NMR spectrum of Figure 2A (left) contains one assigned cross peak for approximately 90% of all its backbone amides, enabling a residue-specific determination of their hydrogen exchange rates in inclusion bodies. Upon exchange in D_2_O buffer for 311 h (Figure 2A, right), many cross peaks show a virtually complete loss of intensity, which is indicative of fast exchange. In contrast, a set of cross peaks of residues 8–25 and 36–43 are still present, which is indicative of slow exchange. The hydrogen exchange was followed over time to get insight into conformational heterogeneity (Figure S2). Individual amides of all residues display a biphasic behavior comprising a very fast and a slow exchanging component (Figure S2). However, for most of the residues, only either the fast or the slow exchanging component is highly populated (Figure 2C; P > 2/3, where P is the population).

The detailed sequence-resolved analysis of the hydrogen exchange data of the major population (Figure 2C) shows that, most of the residues (i.e., 2–6, and 24–95) are only weakly or not protected and are therefore considered to be conformationally disordered. This is indicated by exchange rates faster than 10^1^ h^−1^. In contrast, residues 7–23 display slow exchange rates of 10^−3^ to 10^−4^ h^−1^ and are therefore considered to be involved in hydrogen bonds (Figure 2C). Because the circular dichroism (CD) spectrum is indicative of β-sheet and “random coil” conformation (Figure S3), it is likely that the solvent-protected residues 7–23 contain mainly β-sheet secondary structure. The presence of abundant β-structure is strongly supported by the fiber diffraction data of inclusion bodies of ESAT-6 (Figure 2D). The sharp reflection observed at 4.7 Å is interpreted as the spacing between strands in a β-sheet. The diffuse reflection at approximately 10 Å is interpreted as the spacing between β-sheets. These two reflections are typically observed for amyloid-like fibrils. The circular profiles for these reflections, rather than orthogonal positions for the two reflections, show that the directions of the amyloid-like entities in inclusion bodies are not strongly aligned. To confirm the potential of residues 7–23 to be in a cross-β-sheet amyloid conformation, a synthetic peptide E20 corresponding to residues 6–25 of ESAT-6 was tested for in vitro aggregation. Upon incubation for 3 d at physiological conditions, E20 forms amyloid fibrils as evidenced both by electron microscopy (EM) (Figure S5) and Thio T binding (unpublished data).

To verify that the cross-β-sheet–forming segment of residues 7–23 is the dominant component in the formation of inclusion bodies of ESAT-6, the following mutagenesis experiments were carried out: Within the sequence of ESAT-6, aggregation-prone hydrophobic residues were replaced by the aggregation-interfering Arg [31]. The substitution of L36R, V54R, or I76R did not alter substantially the formation of inclusion bodies. This finding is expected because these residues are located within the disordered region of the inclusion bodies. In contrast, the substitution of F8R, I11R, I18R, or V22R within the β-sheet–forming segment abolished the formation of inclusion bodies (Figure 2E). Similarly, the deletion of the cross-β-sheet–forming segment of residues 7–23 or its replacement by a non–aggregation-prone segment of residues 25–41 of ESAT-6, which forms a helix in the soluble structure of ESAT-6, resulted both in a loss of inclusion body formation (Figure 2E). The lack of soluble expressed ESAT-6 variants comprising the latter substitutions and deletions is attributed to their fast degradation [15]. To confirm that the plasmids of the latter point mutants were functionally conserved, they were mutated back to wild type, which restored the formation of inclusion bodies upon overexpression (Figure S4). Consistent with all our findings, one of the two applied algorithms that predict aggregation-prone segments [32,33] indicates residues approximately 7–23 to be the most-aggregation-prone segment of ESAT-6 (Figure 2C). In summary, residues 7–23 of ESAT-6 in bacterial inclusion bodies form a cross-β-sheet structure characteristic of amyloid-like fibrils [5,10] with the remainder of the amino acid sequence disordered.

### Two Other Inclusion Bodies Also Contain Ordered β-Sheet Structure

Very similar findings are observed for inclusion bodies of BMP2(13–74) and MOG(ECD). Upon exchange in D_2_O buffer for 185 and 285 h, respectively, many cross peaks of the [^15^N,^1^H]-correlation spectra show a virtually complete loss of intensity, which is indicative of fast exchange (Figures 3A and 4A). In contrast, a set of cross peaks of residues 62–70 of BMP2(13–74) and most of the residues 80–94, 102–105, and 110–117 of MOG(ECD), respectively, are still present, which is indicative of slow exchange. Again, the H/D exchange of all residues displayed a biphasic behavior with a very fast and slow exchanging component (Figures 3C, 4C, and S2). The hydrogen exchange data of the major population of inclusion bodies show that residues 14–61 and 68–74 of BMP2(13–74) (Figure 3C), and residues 2–84, 96–100, 109–110, and 119–133 of MOG(ECD) (Figure 4C) are not protected or only weakly protected and may therefore be conformationally disordered. In contrast, residues 62–67 of BMP2(13–74) (Figure 3C) and residues 85–95, 101–108, and 111–118 of MOG(ECD) (Figure 4C) display slow exchange rates of 10^−2^ to 10^−4^ h^−1^ and are therefore considered to be involved in hydrogen bonds. Since diffraction patterns of both inclusion bodies are indicative of cross-β structure (Figures 3D and 4D), it is likely that the solvent-protected segments contain mainly β-sheet secondary structure of cross-β-sheet nature. These findings are further confirmed by the amyloid-like fibril formation of synthetic peptides B13 corresponding to residues 59–71 of BMP2(13–74), and M11 and M18 corresponding to residues 85–95 and 101–118 of MOG(ECD) (Figure S5). Consistent with these data, the combination of the applied algorithms that predict aggregation-prone segments [32,33] indicate residues approximately 62–67 and residues approximately 100–118 to be the most aggregation-prone segments of BMP2(13–74) (Figure 3C) and MOG(ECD) (Figure 4C).

A similar mutagenesis experiment as described above was carried out to verify that the cross-β–forming segment residues 62–67 of BMP2(13–74) and the segments 85–95, 101–108, and 111–118 of MOG(ECD) are the dominant components in the formation of inclusion bodies (Figures 3E and 4E). As expected, the substitutions I32R and L51R of BMP2(13–74) did not alter the formation of inclusion bodies, because the substitutions are located within the disordered segment of the inclusion bodies. In contrast, the substitutions I62R, V63G, V63E, V63R, and L66R of BMP2(13–74) within the β-sheet–forming segment abolished or diminished the formation of inclusion bodies (Figure 3E). Moreover, these variants are present in the soluble fraction of the E. coli lysate upon overexpression (Figure 3E). In particular, the replacement series at position 63 indicate that all kinds of aggregation-interfering residues [31] (i.e., with or without charge, small or large side chains, positive or negative charged) are able to perturb inclusion body formation, which results in the presence of soluble protein (Figure 3E).

To further strengthen the finding that the hydrogen-protected residues 62–67 of BMP2(13–74) are responsible for the formation of inclusion bodies, a plasmid was constructed that codes for residues 61–70 of BMP2(13–74) C-terminally to the protein domain PDZ1 of SAP90, which alone is expressed highly soluble in E. coli (Figure S6). Since the BMP2-tag, denoted as B10, is highly aggregation prone, upon overexpression in E. coli, the fusion protein PDZ1-B10 is present mainly as inclusion bodies (Figure S6). Hence, the hydrogen-protected residues 62–67 appear to be the dominant components in the formation of inclusion bodies.

In the mutagenesis experiment of MOG(ECD), substitutions within the disordered segments did not alter the formation of inclusion bodies, as expected. However, single point substitutions within one β-sheet–forming segment are not sufficient to abolish inclusion body formation, indicating that the presence of one aggregation-prone segment of β-sheet secondary structure is sufficient for inclusion body formation (Figure 4E). Diminished inclusion body formation is observed only with the deletion variants MOG(1–73) and MOG(1–83) which lack all the hydrogen exchange-protected segments (Figure 4E).

In summary, in bacterial inclusion bodies of BMP2(13–74) and MOG(ECD), residues 62–67 of BMP2(13–74) and residues 85–95, 101–108, and 111–118 of MOG(ECD) form cross-β–structure characteristic of amyloid-like fibrils, whereas the remainder of the amino acid sequence is disordered.

### Inclusion Bodies May Contain Amyloid-Like Protofibrils

**Figure 5:**
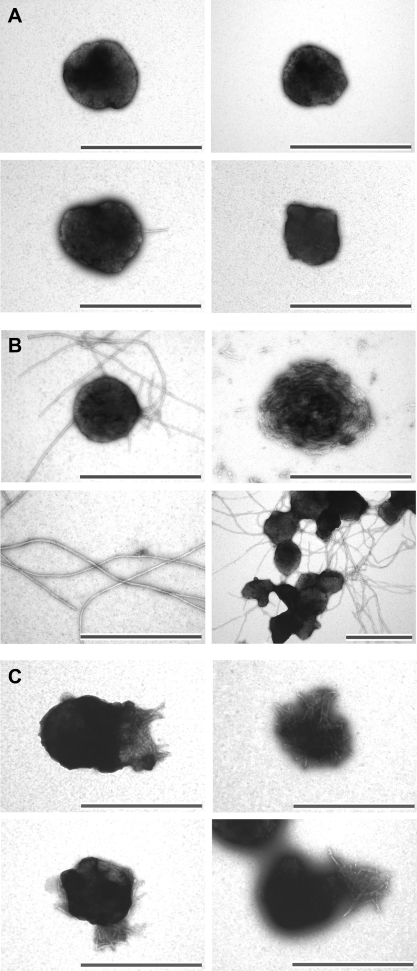
EM of Inclusion Bodies of BMP2(13–74) with Amyloid-Like Fibrils upon Incubation (A) Freshly purified inclusion bodies. They are electron-dense, round aggregates with a diameter approximately 0.5 μm. In some inclusion bodies, short amyloid-like fibrils can be observed. (B) Inclusion bodies after in vitro incubation at 37 °C for 12 h. Fibrils are present from the purified inclusion bodies. (C) Freshly purified inclusion bodies from E. coli cells that overexpressed BMP2(13–74) at 37 °C for 12 h. Fibrils are present from the purified inclusion bodies. Scale bars indicate 1 μm.

Although the inclusion bodies studied here are amyloid-like, they do not display fibrils under EM (Figure 5A). The absence of visible fibrils may be attributed to the high protein concentration in inclusion bodies and to the abundant, stained amorphous segments of the protein (Figures 2, 3, and 4) obscuring the fibrils. An alternative explanation could be that inclusion bodies form too rapidly for fibrils to form from the more immature and flexible protofibrils that have less rigidly linear profiles (see also below). We favor this latter hypothesis because of the following reasons: Varying the amount or time of staining as well as grinding inclusion bodies or treating inclusion bodies with chemical denaturant did not reveal reproducible and significant fibril formation under EM (unpublished data). In contrast, upon incubation of purified inclusion bodies of BMP2(13–74) at 37 °C for 12 h, fibrils appear and grow more prominent with time (Figure 5B).The qualitative analysis of EM shows thereby two scenarios: (1) the fibrils appear to grow from the inclusion bodies (Figure 5A), and (2) the inclusion bodies comprise highly dense bundles of amyloid fibrils (Figure 5B). The apparent growth of fibrils from inclusion bodies can be accelerated by higher temperature (tested up to 50 °C; unpublished data), but is not present in a sample stored for 10 mo at 4 °C (unpublished data). Furthermore, protein overexpression of BMP2(13–74) in E. coli over a period of 12 h under semi-controlled physiological conditions yields inclusion bodies with fibril-like structural entities (Figure 5C). Similarly, upon incubation of purified inclusion bodies of ESAT-6 at room temperature (∼21 °C) for 14 d, amyloid-like fibrils appear under EM (Figure S7). We therefore suggest that inclusion bodies are reminiscent of amyloidogenic protofibrils.

## Discussion

### Common Structural Properties of Inclusion Bodies

Our structural studies of three bacterial inclusion bodies reveal the presence of distinct sequence-specific cross-β-sheet structures surrounded by amorphous disordered segments. The number of cross-β segments is variable (i.e., one to three β-segments in the three proteins studied here). Their lengths are typically around seven to ten residues long, if it assumed that the 16-residue-long solvent-protected segment of ESAT-6 inclusion bodies may be interrupted in the middle. The β-strands in inclusion bodies do not necessary have a β-sheet conformation in the corresponding soluble fold of the protein (Figures 2B, 3B, and 4B). In particular, the segments comprising a β-sheet structure in the inclusion bodies of ESAT-6 and BMP2(13–74) are helical in the corresponding soluble folded conformations (Figures 2B and 3B). However, although the amino acid sequence composition of the various β-strands involved in aggregation are quite heterogeneous, these segments share common properties when compared with nonaggregating sequences. First, they display a low energy when complementing themselves in a pair of β-sheets, as required by the 3DPROFILE algorithm [33], and second, they have a high propensity to form a β-aggregate, as required by the TANGO algorithm [32]. Neither algorithm makes predictions in perfect agreement with the NMR results, but the agreement is good considering that the energy models of the two algorithms are entirely different (Figures 2C, 3C, and 4C).

Hence, in striking contrast to the traditional view of bacterial inclusion bodies as amorphous aggregates, they may contain ordered structural segments of cross-β structure, of which the number, length, and form of its β-sheets are determined by the particular amino acid sequence of the protein. The structures of these regions are thereby probably restricted to one of the possible topologies of cross-β structures [10]. Adjacent to the cross-β-sheet core of the inclusion body aggregates, there may be amorphously disordered segments (Figures 2, 3, and 4), or also folded domains [34,35].

### Evolutionary Pressure against Amyloid Aggregates

Under the assumption that the observed amyloid-like nature of inclusion bodies (Figures 1–4 and [21]) holds for most of the hundreds of documented bacterial inclusion bodies [36], amyloid aggregation appears to be a common property of protein segments and consequently is observed in both eukaryotes and prokaryotes [2]. Within the framework of this hypothesis, it has been suggested that there must be evolved strategies against amyloid formation, which include both quality control mechanisms through molecular chaperones as well as sequence-based prevention of amyloid aggregation [31,32,37,38]. The suggested evolutionary pressure on the protein's amino acid sequence composition against amyloid-like aggregation is evident from our present inclusion body study. The amino acid sequence of over 80% of ESAT-6, 90% of BMP2(13–74), and approximately 75% of MOG(ECD) are disordered in inclusion bodies (Figures 2C, 3C, and 4C) and hence not aggregation prone. This finding indicates that the majority of residues in the amino acid sequences of all three proteins studied have evolved to disfavor aggregation.

### Bacterial Inclusion Bodies and Human Amyloid Diseases

The formation of amyloid-like inclusion bodies of E. coli may also provide insights into fundamental mechanisms of amyloid aggregation-related pathologies such as Alzheimer disease and prion diseases [17]. In these so-called amyloid diseases, a soluble protein or peptide alters its state via conformational intermediates into amyloid fibrils [1]. Some of the intermediates, rather than the amyloid fibrils themselves, have been proposed to be the most toxic amyloid entities [2,39]. These intermediates might include protofibrils. The data presented here and by others suggest that inclusion bodies exhibit structural and some biological properties of protofibrillar amyloid aggregates [40,41] in that inclusion bodies are amyloid-like in structure, are able to mature into amyloid-like fibrils with time (Figure 5), and show amyloid-like “cytotoxicity” when administered to an eukaryotic cell line [42]. Furthermore, we show in Figure S8, that the amyloidogenic mouse prion protein and fragments thereof form amyloid-like inclusion bodies when expressed in E. coli. Despite the similar biological and structural properties of inclusion bodies and protofibrils or fibrils associated with pathological conditions, inclusion bodies are not toxic to its host E. coli and are believed rather to be a detoxification mechanism [43]. The lack of toxicity of inclusion bodies is in agreement with the lack of toxicity of amyloid β-protein (Aβ) amyloids and other amyloid fibrils when administered to a dermal cell line [44]. These findings may support the hypothesis that the toxic species in amyloid diseases is a small oligomer rather than the amyloid fibrils [45–47].

Our studies suggest a tight structural link between amyloid aggregates associated with human amyloid diseases, inclusion bodies of the aggresome [48], and inclusion body formation in E. coli. In addition, these structural studies of bacterial inclusion bodies extend the possible structural landscape of every protein: each protein may exist, not only in an unfolded or folded state, but, by containing at least one amino acid segment that is capable of participating in a sequence-specific, ordered, cross-β-sheet aggregated state, may also exist in an amyloid-like aggregate. The process of protein aggregation can thus be viewed as a primitive folding mechanism, resulting in a defined, aggregated conformation with each aggregated protein having its own distinctive properties.

## Materials and Methods

### Cloning and expression of proteins and variants thereof.

The plasmids of ESAT-6 (M. tuberculosis) is a kind gift of Dr. Jeffery S. Cox (University of California, San Francisco), the plasmid of BMP2 (human) is a kind gift of Dr. Senyon Choe (Salk Institute), and the plasmid of MOG (rat) is a kind gift of Dr. Nancy Ruddle (Yale University) and Dr. Christopher Linington (University of Aberdeen). The DNAs encoding the ESAT-6 and BMP2(13–74) were cloned into the pET21a(+) vector. The DNA encoding the extracellular domain of MOG (MOG(ECD)) was cloned into the pQE-12 vector with and without a C-terminal His-tag [28]. All the experiments throughout this work was done with His-tagged MOG(ECD), since both His-tagged and His-tag–free MOG(ECD) form inclusion bodies with a very similar H/D exchange pattern (unpublished data). All site-directed mutants were generated with the appropriate nucleotide modification by PCR and sequenced for accuracy. All proteins were grown in BL21(DE3) cells at 37 °C, and were induced at optical density at 600 nm (OD_600_) = 1.0 for 3 h by addition of 1 mM isopropyl β-d-thiogalactoside. The isotopically enriched (^15^N/^13^C) samples were prepared by growing cells in M9 medium containing ^15^NH_4_Cl/ [^13^C] glucose. For the overexpression of BMP2(13–74) over a time of 12 h, the pH of the medium was adjusted every 4 h.

### Inclusion body preparation.

The cells from each 500 ml of medium were harvested by centrifugation, resuspended in 20 ml of buffer A (10 mM Tris, 1 mM EDTA, 25 mM dithiothreitol [DTT] [pH 7.5]), and lysed by sonication on ice (5 min × 2, 50% amplitude, Sonic Dismembrator Model 500, Fisher Scientific). Inclusion bodies were sedimented by centrifugation at 10,000*g* for 10 min at 4 °C, and washed three times with the same buffer. See Figure S9 highlighting the individual steps of the purification protocol.

### Thioflavin T binding.

Inclusion body sample was diluted in 500 μl of buffer A to a final concentration of 20 μM. The solution was mixed with 5 μl of 2 mM Thio T prepared in the same buffer. Fluorescence was measured immediately after addition of Thio T. The experiment was measured on a spectrofluorimeter (Photon Technology International) with excitation at 450 nm and emission at 485 nm. A rectangular 10-mm quartz microcuvette was used. As reference, α-synuclein fibrils were measured as follows: a 14-μl aliquot of aged α-synuclein sample (10 mg/ml protein, PBS [pH 7.4]) was diluted in 500 μl of buffer A to a final concentration of 20 μM. The solution was mixed with 5 μl of 2 mM Thio T prepared in the same buffer.

### Congo Red birefringence.

The CR staining was performed using the diagnostic amyloidal stain kit HT60 from Sigma. Briefly, purified inclusion bodies in buffer A were pelleted by centrifuge at 16,000*g* and washed once by distilled water. The inclusion bodies were then placed in 500 μl of alkaline sodium chloride solution for 20 min, with continuous vortexing. The continuous vortexing enhances the uniform mixing of all inclusion bodies particles in solution. The mixture was then centrifuged briefly with 16,000*g*, and pellets were taken to stain with alkaline CR solution for 20 min, with continuous vortexing. The mixtures were centrifuged briefly at 16.000*g*, and pellets were washed twice with 500 μl of 20% ethanol. The pellets were resuspended in PBS and then spread evenly onto glass slides and air dried at room temperature. The slides were analyzed using a microscope equipped with two polarizers equipped with a charge-coupled device (CCD) camera.

### Sequential NMR assignment.

Nearly complete sequence-specific assignment of the backbone H^N^/N cross peaks in the [^15^N,^1^H]-HMQC spectra were obtained for all three proteins using the triple-resonance experiments HNCACB [49] and (H)N(COCA)NH [50] applied to ^13^C,^15^N-labeled inclusion bodies solubilized in dimethyl sulfoxide-d6 (d_6_-DMSO) containing 0.05% trifluoroacetic acid (TFA) and 25 mM DTT.

### H/D exchange.

Homogenously ^15^N-labeled inclusion bodies of BMP2 (13–74), ESAT-6, and MOG(ECD) were used for the H/D-exchange studies. To start the H/D exchange, the inclusion bodies solution (0.5 ml, 200 μM) was centrifuged at 13 000*g* for 3 min, the separated inclusion bodies pellet was washed with D_2_O twice, then resuspended in 0.5 ml of D_2_O and kept at 4 °C for H/D exchange. Right before each NMR measurement, the H/D-exchanged inclusion bodies were sedimented at 13,000*g* for 3 min and dissolved in d6-DMSO containing 0.05% TFA and 25 mM DTT. Immediately after dissolving the inclusion bodies, the [^15^N,^1^H]-correlation spectra (HMQC) were measured for 5 min. Residues that display high intrinsic exchange rates in DMSO were determined by the addition of H_2_O followed by the measurement of a series of 2-D spectra. Using this control measurement, some residues were excluded from the H/D-exchange data analysis. To confirm that during the H/D-exchange measurement the inclusion bodies were preserved in their structural properties, EM and Thio T binding studies were performed at day 0 and day 15 (Figures 5, S7, and S10). In addition, an H/D-exchange experiment with an exchange time of 1 h of 15-d-old inclusion bodies of ESAT-6 was measured, which showed no qualitative difference when compared with freshly prepared inclusion bodies thereof (unpublished data). Since EM, Thio T, and H/D-exchange measurements of fresh (0 day) and 2-wk-old inclusion bodies show close resemblance, their integrity over the entire H/D-exchange experiment is assumed. The spectra were analyzed using the programs PROSA [51] and CARA [52]. At least two independent experiments were carried out for each protein system.

### X-ray diffraction.

Pelleted inclusion bodies were resuspended in a minimal amount of water. Then 5 μl of the suspension were pipetted between two fire-polished glass rods and left to dry for 2–3 d. The dried material was placed in an X-ray beam at room temperature for a 5-min exposure. A rotating anode generator (Rigaku FR-E) and imaging plate detector (RaxisIV++) were used for the data collection. Two independent experiments were carried out for each protein system.

### SDS-PAGE.

After cell lysis and centrifugation, soluble and insoluble fractions were separated. The insoluble fraction was resuspended in buffer A in the same amount as the soluble fraction. To compare the protein amount in each fraction, 5 μl of solution was taken from either the soluble or the resuspended insoluble fraction, and was mixed with 10 μl of 10 M UREA and 5 μl of NuPAGE LDS sample buffer (Invitrogen). Each sample was boiled for 3 min before loaded to NuPAGE 12% Bis-Tris gel (Invitrogen). Proteins were visualized by Coomassie Blue staining (0.1% Coomassie Blue, 10% acetic acid, 40% methanol). At least two independent experiments were carried out for each protein system.

To verify the identity of the bands on the SDS gel, that were present only upon overexpression of wild-type or mutant BMP2(13–74) fragments, the bands of interest were excised and digested with trypsin. After digestion, peptides were extracted and analyzed by matrix-assisted laser desorption/ionization (MALDI)-mass spectrometry (MS). Mass spectrometry was measured on an Applied Biosystems Voyager DE-STR instrument in linear mode (expected mass accuracy 0.5% or better). Alpha-cyano-hydroxybenzoic acid was used as the matrix. An average of 100 spectra were summed for each measurement. For both set of bands from inclusion body samples and the soluble samples, masses corresponding to the expected size of the wild-type and mutant BMP2(13–74) were observed. Observation of the masses not only verified the identity of the bands but also proved the presence of the mutations.

### Circular dichroism.

The inclusion body preparation of ESAT-6 was diluted into buffer A to a final concentration of 20 μM. The experiment was measured on a CD spectrophotometer (BioLogic) from 200 nm to 260 nm. A 1-mm-thick quartz microcuvette was used.

### Electron microscopy.

The inclusion body or peptide sample was diluted into buffer A to a final concentration of 50 μM, spotted on a glow-discharged, carbon-coated Formvar grid (Electron Microscopy Sciences), incubated for 5 min, washed with distilled water, and then stained with 1% (w/v) aqueous uranyl formate solution. Uranyl formate solutions were filtered through 0.2-μm sterile syringe filters (Corning) before use. EM analysis was performed using a JEOL JEM-100CXII electron microscope at 80 kV with nominal magnification of 48,000×. Images were recorded digitally by using the SIS Megaview III imaging system. At least two independent experiments were carried out for each sample.

### Peptide fibril formation.

Peptides were synthesized either by the Salk Institute (E20) or Tufts University peptide facility (B13, M11, and M18) using the solid-phase peptide synthesis approach. Solid peptides were dissolved in buffer A to a final concentration of 500 μM, and the pH was adjusted to 7.5. The peptide solution was centrifuged at 13 000*g* for 10 min, and the supernatant was incubated at 37 °C with stirring for 3 d before observation. The fibril formation was monitored by EM and Thio T binding.

## Supporting Information

Figure S1 Amyloid-Like Properties in Three Bacterial Inclusion Bodies CR staining and birefringence of inclusion bodies of BMP2(13–74), ESAT-6, and MOG(ECD) are indicative of amyloid formation. The pictures on the left-hand side represent the bright-field microscope images with 10× resolution. The same sections are shown on the right-hand side under cross-polarized light with 10× magnification, respectively.
(8.91 MB TIF)

Figure S2 NMR Exchange Curves of ESAT-6, BMP2(13–74), and MOG(ECD) as Indicated The evolutions of the peak intensities versus the H/D-exchange time are shown. Smooth solid lines represent the monoexponential fits of the raw data after an initial drop of the intensities within the first measurement points. Data for the residues S77 (yellow), K57 (green), A9 (red), and T23 (cyan) of ESAT-6 are shown. In addition, the exchange data for residues F41 (yellow), V21 (green), V67 (red), and I62 (cyan) of BMP2(13–74) are shown. Furthermore, A24 (yellow), G42 (green), A86 (red), and F104 (cyan) of MOG(ECD) are shown. For all the exchange curves, a biphasic behavior is observed. After an initial drop of the intensities within the first measurement points, a slow exponential decay is manifested. The relative population *P*(F) of the two exchange species is residue-dependent and listed in Figures 2C, 3C, and 4C. The initial drop is attributed to fast H/D exchange reminiscent of an amorphous structure, whereas the slow exponential decay is attributed to slow H/D exchange reminiscent of a homogenous conformational species with hydrogen bond formation.
(1.14 MB TIF)

Figure S3 Circular Dichroism of Inclusion Bodies of ESAT-6 Indicative of β-Sheet Conformation The mean helical ellipticity [θ] is shown versus the wavelength of the light source in nanometers.
(613 KB TIF)

Figure S4 Control Experiment for the Mutagenesis of ESAT-6 Coomassie-stained SDS-polyacrylamide gels were obtained from soluble (s) and insoluble (i) fractions of lysates of E. coli cells expressing wild-type ESAT-6 constructs that have been back-mutated from the variants F8R, I11R, I18R, and V22R to wild-type. Since all back-mutated plasmid constructs show wild-type–like inclusion body formation, the plasmids of F8R, I11R, I18R, and V22R used in Figure 2E are assumed to be functional. Hence, these controls show that the amino acid substitution F8R, I11R, I18R, and V22R abolish inclusion body formation.
(125 KB TIF)

Figure S5 Amyloid Fibril Formation of Peptides B13, E20, M11, and M18 The peptides correspond to the amino acid sequence segments of BMP2(59–71), ESAT-6(6–25), MOG(85–95), and MOG(101–118) that are involved in cross-β-sheet structure in inclusion bodies. Upon incubation of 3 d, all peptides form amyloid fibrils. Transmission EM was performed of negative-stained, aged peptide samples. Scale bars indicate 1 μm. The amyloid fibril formation of all peptides were also confirmed by Thio T binding (unpublished data).
(2.25 MB TIF)

Figure S6 Residues 61–70 of BMP(13–74) Move the Otherwise Solubly Expressed PDZ1 Domain of SAP90 into Inclusion Bodies When Fused C-Terminally to PDZ1 Coomassie-stained SDS-polyacrylamide gels were obtained from soluble (s) and insoluble (i) fractions of lysates of E. coli cells expressing wild-type PDZ1 and a fusion construct, denoted PDZ1-B10, with residues 61–70 of BMP2 located C-terminally to the PDZ1 domain of SAP90. The 10- and 14-kDa molecular weight standards are labeled.
(1.58 MB TIF)

Figure S7 EM of Inclusion Bodies of ESAT-6 with Amyloid-Like Fibrils upon Incubation (A) Freshly purified inclusion bodies of ESAT-6 are electron-dense, round aggregates with diameter approximately 0.5 μm.
(B) Inclusion bodies after in vitro incubation at room temperature (∼21 °C) for 14 d. Fibrils are present from the purified inclusion bodies.
Scale bars indicate 1 μm.
(2.39 MB TIF)

Figure S8 Amyloid-Like Character of Bacterial Inclusion Bodies of Mouse Prion Protein and Fragments Thereof: PrP(23–231), PrP(90–231), and PrP(121–231) CR staining and birefringence under cross-polarized light with 10× magnification are shown as indicated.
(7.93 MB TIF)

Figure S9 The Purification Procedures of Inclusion Bodies of ESAT-6, BMP2(13–74), and MOG(ECD) as Indicated Coomassie-stained SDS-polyacrylamide gels were obtained from (1) E. coli cells before induction, (2) E. coli cells after induction, (3) soluble fraction of the cell lysate, (4) insoluble fraction of the cell lysate, and (5) purified inclusion bodies. The molecular weight standards are labeled under “M.”
(2.67 MB TIF)

Figure S10 Control Experiments That Show the Preservation of the Properties of Inclusion Bodies during the H/D-Exchange Experiment (A) Thio T binding assay before (day 0) and after the H/D-exchange experiment (day 15) as indicated.
(B) Electron micrograms of the inclusion bodies after the H/D exchange experiment at day 15 which are similar to fresh inclusion bodies (Figures 5 and S7). See also Materials and Methods.
(872 KB TIF)

## Abbreviations

CD: circular dichroism
CR: Congo red
DTT: dithiothreitol
ECD: extracellular domain
EM: electron microscopy
H/D: hydrogen/deuterium
MOG: myelin oligodendrocyte glycoprotein
NMR: nuclear magnetic resonance
TFA: trifluoroacetic acid
Thio T: thioflavin T

## References

[pbio-0060195-b001] Selkoe DJ (2003). Folding proteins in fatal ways. Nature.

[pbio-0060195-b002] Dobson CM (2003). Protein folding and misfolding. Nature.

[pbio-0060195-b003] Kelly JW (2005). Attacking amyloid. N Engl J Med.

[pbio-0060195-b004] Ventura S (2005). Sequence determinants of protein aggregation: tools to increase protein solubility. Microb Cell Fact.

[pbio-0060195-b005] Sunde M, Serpell LC, Bartlam M, Fraser PE, Pepys MB (1997). Common core structure of amyloid fibrils by synchrotron X-ray diffraction. J Mol Biol.

[pbio-0060195-b006] Kirschner DA, Abraham C, Selkoe DJ (1986). X-ray diffraction from intraneuronal paired helical filaments and extraneuronal amyloid fibers in Alzheimer disease indicates cross-beta conformation. Proc Natl Acad Sci U S A.

[pbio-0060195-b007] Nelson R, Sawaya MR, Balbirnie M, Madsen AO, Riekel C (2005). Structure of the cross-beta spine of amyloid-like fibrils. Nature.

[pbio-0060195-b008] Ritter C, Maddelein ML, Siemer AB, Luhrs T, Ernst M (2005). Correlation of structural elements and infectivity of the HET-s prion. Nature.

[pbio-0060195-b009] Luhrs T, Ritter C, Adrian M, Riek-Loher D, Bohrmann B (2005). 3D structure of Alzheimer's amyloid-beta(1–42) fibrils. Proc Natl Acad Sci U S A.

[pbio-0060195-b010] Sawaya MR, Sambashivan S, Nelson R, Ivanova MI, Sievers SA (2007). Atomic structures of amyloid cross-beta spines reveal varied steric zippers. Nature.

[pbio-0060195-b011] Petkova AT, Ishii Y, Balbach JJ, Antzutkin ON, Leapman RD (2002). A structural model for Alzheimer's beta-amyloid fibrils based on experimental constraints from solid state NMR. Proc Natl Acad Sci U S A.

[pbio-0060195-b012] LeVine H (1999). Quantification of beta-sheet amyloid fibril structures with thioflavin T. Methods Enzymol.

[pbio-0060195-b013] Klunk WE, Jacob RF, Mason RP (1999). Quantifying amyloid by congo red spectral shift assay. Methods Enzymol.

[pbio-0060195-b014] Rousseau F, Schymkowitz J, Serrano L (2006). Protein aggregation and amyloidosis: confusion of the kinds. Curr Opin Struct Biol.

[pbio-0060195-b015] Fink AL (1998). Protein aggregation: folding aggregates, inclusion bodies and amyloid. Fold Des.

[pbio-0060195-b016] Ventura S, Villaverde A (2006). Protein quality in bacterial inclusion bodies. Trends Biotechnol.

[pbio-0060195-b017] Freedman RB, Wetzel R (1992). Protein engineering. Curr Opin Biotechnol.

[pbio-0060195-b018] Chrunyk BA, Evans J, Lillquist J, Young P, Wetzel R (1993). Inclusion body formation and protein stability in sequence variants of interleukin-1 beta. J Biol Chem.

[pbio-0060195-b019] Rinas U, Bailey JE (1992). Protein compositional analysis of inclusion bodies produced in recombinant Escherichia coli. Appl Microbiol Biotechnol.

[pbio-0060195-b020] Przybycien TM, Dunn JP, Valax P, Georgiou G (1994). Secondary structure characterization of beta-lactamase inclusion bodies. Protein Eng.

[pbio-0060195-b021] Carrio M, Gonzalez-Montalban N, Vera A, Villaverde A, Ventura S (2005). Amyloid-like properties of bacterial inclusion bodies. J Mol Biol.

[pbio-0060195-b022] Ignatova Z, Krishnan B, Bombardier JP, Marcelino AM, Hong J (2007). From the test tube to the cell: exploring the folding and aggregation of a beta-clam protein. Biopolymers.

[pbio-0060195-b023] Speed MA, Wang DI, King J (1996). Specific aggregation of partially folded polypeptide chains: the molecular basis of inclusion body composition. Nat Biotechnol.

[pbio-0060195-b024] King J, Haase-Pettingell C, Robinson AS, Speed M, Mitraki A (1996). Thermolabile folding intermediates: inclusion body precursors and chaperonin substrates. FASEB J.

[pbio-0060195-b025] Renshaw PS, Lightbody KL, Veverka V, Muskett FW, Kelly G (2005). Structure and function of the complex formed by the tuberculosis virulence factors CFP-10 and ESAT-6. EMBO J.

[pbio-0060195-b026] Scheufler C, Sebald W, Hulsmeyer M (1999). Crystal structure of human bone morphogenetic protein-2 at 2.7 A resolution. J Mol Biol.

[pbio-0060195-b027] Allendorph GP, Vale WW, Choe S (2006). Structure of the ternary signaling complex of a TGF-beta superfamily member. Proc Natl Acad Sci U S A.

[pbio-0060195-b028] Breithaupt C, Schubart A, Zander H, Skerra A, Huber R (2003). Structural insights into the antigenicity of myelin oligodendrocyte glycoprotein. Proc Natl Acad Sci U S A.

[pbio-0060195-b029] Clements CS, Reid HH, Beddoe T, Tynan FE, Perugini MA (2003). The crystal structure of myelin oligodendrocyte glycoprotein, a key autoantigen in multiple sclerosis. Proc Natl Acad Sci U S A.

[pbio-0060195-b030] Hoshino M, Katou H, Hagihara Y, Hasegawa K, Naiki H (2002). Mapping the core of the beta(2)-microglobulin amyloid fibril by H/D exchange. Nat Struct Biol.

[pbio-0060195-b031] Rousseau F, Serrano L, Schymkowitz JW (2006). How evolutionary pressure against protein aggregation shaped chaperone specificity. J Mol Biol.

[pbio-0060195-b032] Fernandez-Escamilla AM, Rousseau F, Schymkowitz J, Serrano L (2004). Prediction of sequence-dependent and mutational effects on the aggregation of peptides and proteins. Nat Biotechnol.

[pbio-0060195-b033] Thompson MJ, Sievers SA, Karanicolas J, Ivanova MI, Baker D (2006). The 3D profile method for identifying fibril-forming segments of proteins. Proc Natl Acad Sci U S A.

[pbio-0060195-b034] Garcia-Fruitos E, Gonzalez-Montalban N, Morell M, Vera A, Ferraz RM (2005). Aggregation as bacterial inclusion bodies does not imply inactivation of enzymes and fluorescent proteins. Microb Cell Fact.

[pbio-0060195-b035] Sambashivan S, Liu Y, Sawaya MR, Gingery M, Eisenberg D (2005). Amyloid-like fibrils of ribonuclease A with three-dimensional domain-swapped and native-like structure. Nature.

[pbio-0060195-b036] Deuerling E, Schulze-Specking A, Tomoyasu T, Mogk A, Bukau B (1999). Trigger factor and DnaK cooperate in folding of newly synthesized proteins. Nature.

[pbio-0060195-b037] Monsellier E, Chiti F (2007). Prevention of amyloid-like aggregation as a driving force of protein evolution. EMBO Rep.

[pbio-0060195-b038] Richardson JS, Richardson DC (2002). Natural beta-sheet proteins use negative design to avoid edge-to-edge aggregation. Proc Natl Acad Sci U S A.

[pbio-0060195-b039] Lambert MP, Barlow AK, Chromy BA, Edwards C, Freed R (1998). Diffusible, nonfibrillar ligands derived from Abeta1–42 are potent central nervous system neurotoxins. Proc Natl Acad Sci U S A.

[pbio-0060195-b040] Chimon S, Ishii Y (2005). Capturing intermediate structures of Alzheimer's beta-amyloid, Abeta(1–40), by solid-state NMR spectroscopy. J Am Chem Soc.

[pbio-0060195-b041] Kayed R, Head E, Thompson JL, McIntire TM, Milton SC (2003). Common structure of soluble amyloid oligomers implies common mechanism of pathogenesis. Science.

[pbio-0060195-b042] Gonzalez-Montalban N, Villaverde A, Aris A (2007). Amyloid-linked cellular toxicity triggered by bacterial inclusion bodies. Biochem Biophys Res Commun.

[pbio-0060195-b043] Gonzalez-Montalban N, Carrio MM, Cuatrecasas S, Aris A, Villaverde A (2005). Bacterial inclusion bodies are cytotoxic in vivo in absence of functional chaperones DnaK or GroEL. J Biotechnol.

[pbio-0060195-b044] Maji SK, Schubert D, Rivier C, Lee S, Rivier JE (2008). Amyloid as a depot for the formulation of long-acting drugs. PLoS Biol.

[pbio-0060195-b045] Hardy J, Selkoe DJ (2002). The amyloid hypothesis of Alzheimer's disease: progress and problems on the road to therapeutics. Science.

[pbio-0060195-b046] Glabe CC (2005). Amyloid accumulation and pathogenesis of Alzheimer's disease: significance of monomeric, oligomeric and fibrillar Abeta. Subcell Biochem.

[pbio-0060195-b047] Ross CA, Poirier MA (2004). Protein aggregation and neurodegenerative disease. Nat Med.

[pbio-0060195-b048] Kopito RR (2000). Aggresomes, inclusion bodies and protein aggregation. Trends Cell Biol.

[pbio-0060195-b049] Salzmann M, Wider G, Pervushin K, Senn H, Wuthrich K (1999). TROSY-type triple-resonance experiments for sequential NMR assignments of large proteins. J Am Chem Soc.

[pbio-0060195-b050] Bracken C, Palmer AG, Cavanagh J (1997). (H)N(COCA)NH and HN(COCA)NH experiments for 1H-15N backbone assignments in 13C/15N-labeled proteins. J Biomol NMR.

[pbio-0060195-b051] Guntert P, Dotsch V, Wider G, Wuthrich K (1992). Processing of multi-dimensional NMR data with the new software PROSA. J Biomol NMR.

[pbio-0060195-b052] Keller R (2004). The computer aided resonance assignment tutorial.

[pbio-0060195-b053] Cuff JA, Clamp ME, Siddiqui AS, Finlay M, Barton GJ (1998). JPred: a consensus secondary structure prediction server. Bioinformatics.

[pbio-0060195-b054] Gasteiger E, Hoogland C, Gattiker A, Duvaud S, Wilkins MR, Walker JM (2005). Protein identification and analysis tool on the EXPASY server. The proteomics protocols handbook.

[pbio-0060195-b055] Eisenberg D, Schwarz E, Komaromy M, Wall R (1984). Analysis of membrane and surface protein sequences with the hydrophobic moment plot. J Mol Biol.

